# Prevalence of ketosis, ketonuria, and ketoacidosis during liberal glycemic control in critically ill patients with diabetes: an observational study

**DOI:** 10.1186/s13054-016-1462-7

**Published:** 2016-09-15

**Authors:** Nora Luethi, Luca Cioccari, Marco Crisman, Rinaldo Bellomo, Glenn M. Eastwood, Johan Mårtensson

**Affiliations:** 1 Department of Intensive Care, Austin Hospital, 145 Studley Rd, Heidelberg, 3084 VIC Australia; 2 Department of Intensive Care Medicine, Lucerne Cantonal Hospital, Lucerne, Switzerland; 3 Department of Perioperative Medicine, Intensive Care and Emergency, Cattinara Hospital, Trieste University School of Medicine, Trieste, Italy; 4 Australian and New Zealand Intensive Care Research Centre (ANZIC-RC), Department of Epidemiology and Preventive Medicine, Monash University, Melbourne, VIC Australia; 5 Department of Anaesthesia and Intensive Care Medicine, Karolinska University Hospital, Department of Physiology and Pharmacology, Karolinska Institutet, 171 77 Stockholm, Sweden

**Keywords:** Ketone bodies, 3-beta-hydroxybutyrate, Diabetes mellitus, Critical illness, Permissive hyperglycemia, Adults

## Abstract

**Background:**

It is uncertain whether liberal glucose control in critically ill diabetic patients leads to increased ketone production and ketoacidosis. Therefore, we aimed to assess the prevalence of ketosis, ketonuria and ketoacidosis in critically ill diabetic patients treated in accordance with a liberal glycemic control protocol.

**Methods:**

We performed a prospective observational cohort study of 60 critically ill diabetic patients with blood and/or urine ketone bodies tested in ICU. All patients were treated according to a liberal glucose protocol targeting a blood glucose level (BGL) between 10 and 14 mmol/l in a single tertiary intensive care unit in Australia. We measured quantitative bedside blood 3-beta-hydroxybutyrate (β-OHB) and semi-quantitative urine ketones on ICU admission and daily during ICU stay, for a maximum of 10 consecutive days.

**Results:**

Median blood β-OHB level on admission was 0.3 (0.1, 0.8) mmol/l. Ketoacidosis was rare (3 %), but some level of ketosis (β-OHB ≥0.6 mmol/l) was found in 38 patients (63 %) early during their ICU stay. However, there was no significant difference in prevalence or severity of ketonemia and ketonuria among patients with BGL above (permissive hyperglycemia) or below 10 mmol/l. On multivariable linear regression analysis there was no association between blood ketone levels and BGL, HbA1c, lactate levels, hematocrit, catecholamine infusion or APACHE III score. In contrast, blood ketone levels tended to be higher after cardiopulmonary bypass surgery (*P* = 0.06).

**Conclusions:**

Liberal glycemic control in critically ill diabetic patients does not appear to be associated with a high prevalence of ketoacidosis or ketonemia. Moreover, ketosis is typically present on admission and resolves rapidly. Finally, cardiopulmonary bypass surgery may be an important trigger of ketone body production.

**Trial registration:**

Australian New Zealand Clinical Trials Registry (ACTRN12615000216516; trial registration date 5 March 2015).

**Electronic supplementary material:**

The online version of this article (doi:10.1186/s13054-016-1462-7) contains supplementary material, which is available to authorized users.

## Background

Current guidelines recommend using insulin to maintain a blood glucose level (BGL) between 6 and 10 mmol/l in critically ill patients [1–4]. However, controlling glucose within this target range is associated with a relatively high rate of hypoglycemia [5] and increased mortality [6] in diabetic patients especially if adapted to chronic hyperglycemia (glycated hemoglobin A1c [HbA1c] >7 %). Therefore, a more liberal glycemic approach (target BGL between 10 and 14 mmol/l) may be safer in patients with diabetes. Recent pilot studies have demonstrated that such "permissive hyperglycemia" effectively reduces the need for exogenous insulin administration [7]. Consequently, in response to the introduction of a liberal glucose protocol in diabetic patients, the incidence of both absolute hypoglycemia (BGL <3.9 mmol/l) and potentially harmful relative hypoglycemia (an acute and marked decrease in glycaemia) may be reduced.

However, whether such liberal strategies lead to relative insulin deficiency, accelerated ketone body production and induce ketoacidosis is uncertain. In addition to insulin deficiency, several other factors may stimulate ketogenesis via beta-oxidation of free fatty acids (FFA) in critically ill patients. Such factors include fasting (a ubiquitous state in patients admitted to the intensive care unit [ICU]), increased secretion of counter-regulatory hormones (glucagon, adrenaline, growth hormone and cortisol), administration of exogenous catecholamines (adrenaline and noradrenaline) [8] and administration of parenteral nutrition containing medium-chain triglycerides [9]. Furthermore, entry of acetyl coenzyme A into the Krebs cycle may be impaired during critical illness due to reduced oxaloacetate levels. Consequently, more acetyl coenzyme will be used for synthesis of ketone bodies [10]. Similarly, elevated levels of deaminated amino acids may feed ketogenesis. Moreover, although the mechanism is less clear, alkalosis appears to stimulate ketogenesis, particularly in diabetic subjects [11]. Finally, elevated blood ketone levels have been observed following cardiopulmonary bypass surgery, especially when performed under hypothermia [12]. Although, many of these factors may co-exist in the ICU setting, their combined impact on ketone body production is unknown.

Accordingly, we conducted a prospective observational study to explore the incidence of ketosis, ketonuria and ketoacidosis during liberal glucose management of critically ill diabetic patients. In addition, we aimed to assess the association of chronic glycaemia (HbA1c), acute glycaemia, illness severity, alkalosis, catecholamine infusion and cardiopulmonary bypass surgery with blood ketone levels on ICU admission in such patients.

## Methods

### Data collection

We performed a prospective observational study in all diabetic patients admitted to a single tertiary adult ICU between 30 August and 17 November 2015. All patients were treated in accordance with our local protocol for diabetic patients targeting a blood glucose levels (BGL) between 10 and 14 mmol/l and in which BGLs above 14 mmol/l were treated with intravenous or subcutaneous insulin. The Austin Health Human Research Ethics Committee approved the study (approval number LNR/14/Austin/487, HREC 131217 and 120712) with a waiver for informed consent. The study was performed in accordance with the ethical standards laid down in the 1964 Declaration of Helsinki and its later amendments.

For the purpose of this study, we excluded patients admitted with diabetic ketoacidosis (DKA) or a hyperglycemic hyperosmolar state (HHS). We collected demographic data, ICU admission diagnoses, type of diabetes, insulin dependency, chronic medication and Acute Physiology and Chronic Health Evaluation (APACHE) III score. We obtained daily data on use of renal replacement therapy (RRT), incidence of acute liver failure, rate of adrenaline and noradrenaline infusion, type and rate of enteral or parenteral nutrition and insulin administration.

### Blood and urine sample collection and analysis

We collected arterial blood gas (ABG) data and spot urine samples for measurement of blood and urine ketone levels as soon as possible after ICU admission. Thereafter, we performed daily ketone measurement early in the morning, which likely represents peak ketone levels [13] and simultaneously tested urine samples for ketonuria until ICU discharge or up to a maximum of 10 days. We measured blood β-hydroxybutyrate (β-OHB) using the Freestyle Optium Xceed point-of-care meter (Abbott Diabetes Care Inc., Maidenhead, UK). Simultaneously, we measured hematocrit since previous data suggest that point-of-care platforms may overestimate β-OHB at subnormal hematocrit levels [14]. Ketonuria was semi-quantified using Combur-test® strips (Roche Diagnostics, Rotkreuz, Switzerland). ABG samples were analyzed using the Radiometer ABL825 blood gas analyzer (Radiometer Medical A/S, Brønshøj, Denmark). We analysed HbA1c on ICU admission at our central laboratory, using COBAS INTEGRA® 800 (Roche Diagnostics, Indianapolis, IN, USA).

### Definitions

We categorized ketosis according to blood β-OHB levels as absent (<0.6 mmol/l), mild (0.6–1.5 mmol/l), moderate (1.6–3 mmol/l) or marked (>3 mmol/l). Ketonuria was semi-quantified as mild, moderate or marked for urine ketone levels of 1–4 mmol/l, 5–14 mmol/l and ≥15 mmol/l, respectively. The presence of ketoacidosis was assessed using both definitions suggested by the American Diabetes Association (ADA) and the Joint British Diabetes Society (JBDS) [15, 16].

### Statistical analysis

We analyzed data using STATA® version Stata/SE 13.1 (Stata Corp., College Station, TX, USA). Continuous variables were expressed as median (interquartile range [IQR]) and categorical variables as n (%). We compared continuous data using the Mann-Whitney *U* test (two groups) or the Kruskal-Wallis test (multiple groups). For categorical variables, we used the chi-squared test or the Fisher’s exact test. The Spearman’s rank correlation coefficient was calculated to assess correlations. Changes over time for BGLs and blood ketone levels during permissive hyperglycemia (BGL 10–14 mmol/l) were tested by repeated measures analysis of variance (RM-ANOVA) using ICU day as the repeated measures variable. For comparison of these variables’ change over time in patients who did and did not receive insulin, an interaction variable (between category [insulin therapy, yes vs. no] and time) was introduced in the RM-ANOVA model. We used multivariable linear regression analysis to assess the relationship between blood ketone levels on ICU admission and the following variables: APACHE III score (tertiles), ongoing catecholamine infusion (yes vs. no), HbA1c, admission BGL, cardiopulmonary bypass surgery (yes vs. no), admission hematocrit and admission blood lactate level. Since insulin therapy may impact both ketogenesis and BGLs we restricted the multivariable linear regression analysis to patients not receiving insulin. A two-sided *P* value <0.05 was considered statistically significant.

## Results

### Baseline characteristics

We studied 60 consecutive critically ill diabetic patients (17 [28 %] females) with a median (IQR) age of 66 (60, 74) years and a median APACHE III score of 60 (46, 76). Overall, 57 (95 %) patients had type 2 diabetes of whom 24 (42 %) were insulin-dependent prior to ICU admission. Two patients were treated with a sodium-glucose cotransporter-2 inhibitor before ICU admission; none developed ketosis or ketonuria in ICU. The majority (42 %) of patients were admitted after cardiovascular surgery, of which 22 patients underwent cardiopulmonary bypass surgery (during normothermia in all 22 patients). Three (5 %) patients were admitted with acute liver failure and one with acute alcohol intoxication. Four patients had a history of chronic alcohol abuse.

A total of 46 (77 %) patients had a peak BGL ≥10 mmol/l during ICU admission. Compared to patients with a peak BGL <10 mmol/l (n = 14), patients with a peak BGL ≥10 mmol/l were younger (*P* = 0.02), had higher HbA1c (7.0 [6.2, 7.8] % vs. 6.2 [5.9, 6.5] %, *P* = 0.009) and greater admission BGL (*P* = 0.01). Moreover, a higher proportion of patients with peak BGL ≥10 mmol/l were prescribed chronic insulin therapy (23 [50 %] patients vs. 1 [7 %] patient, *P* = 0.007). However, we observed no significant differences in APACHE III score, admission blood ketone levels, admission blood lactate levels or hematocrit levels between the two groups. A total of 21 (46 %) patients with peak BGL ≥10 mmol/l and two (14 %) patients with peak BGL <10 mmol/l received catecholamine infusion on admission (*P* = 0.06). Admission alkalosis (pH >7.45) was relatively uncommon and only six (10 %) patients received enteral or parenteral nutrition on admission. Median length of stay in ICU (3.7 [2.2, 6.2] days) and in hospital (11 [6.7, 24] days) as well as ICU (8 %) and hospital (10 %) mortality was similar in the two groups (Table 1).

**Table 1:** Baseline characteristics and outcomes for all diabetic patients and according to peak blood glucose level

Characteristic	All patients	Peak BGL ≥10 mmol/l	Peak BGL <10 mmol/l	*P* value
Number of patients	60 (100)	46 (77)	14 (23)	
Age, years	66 (60, 74)	65 (56, 71)	74 (65, 80)	0.02
Female sex, n (%)	17 (28)	15 (33)	2 (14)	0.31
Weight, kg (n = 58)	84 (74, 100)	84 (78, 104)	83 (74, 100)	0.39
BMI, kg/m^2^ (n = 40)	31 (27, 34)	32 (28, 35)	28 (24, 31)	0.11
APACHE III score	60 (46, 76)	59 (46, 75)	62 (48, 79)	0.71
Type 1 diabetes, n (%)	3 (5)	3 (7)	0	
Type 2 diabetes, n (%)	57 (95)	43 (93)	14 (100)	1.00
Type 2 diabetes treatment, n (%)				0.007
Diet only	8 (13)	5 (11)	6 (43)	
Oral agent(s) only	25 (42)	18 (39)	7 (50)	
Insulin only	11 (18)	10 (22)	1 (7)	
Insulin + oral agent(s)	13 (22)	13 (28)	0	
Non-operative admission diagnosis, n (%)				0.59
Cardiovascular	6 (10)	5 (26)	1 (14)	
Sepsis	6 (10)	5 (26)	1 (14)	
Respiratory	6 (10)	3 (16)	3 (43)	
Renal/metabolic	6 (10)	5 (26)	1 (14)	
Other	2 (3)	1 (5)	1 (14)	
Operative admission diagnosis, n (%)				0.09
Cardiovascular	25 (42)	20 (74)	5 (71)	
Gastrointestinal	6 (10)	6 (22)	1 (14)	
Other	3 (5)	1 (4)	1 (14)	
HbA1c on ICU admission, %	6.7 (6.0, 7.7)	7.0 (6.2, 7.8)	6.2 (5.9, 6.5)	0.009
Blood glucose on ICU admission, mmol/l	9.3 (7.6, 12)	10 (7.8, 13)	8.1 (7.4, 9.0)	0.01
Blood ketones on ICU admission, mmol/l	0.3 (0.1, 0.8)	0.3 (0.1, 0.8)	0.4 (0.2, 0.6)	0.99
Ketosis on ICU admission, n (%)	23 (39)	17 (38)	6 (43)	0.12
Ketonuria on ICU admission, n (%) (n = 57)	8 (14)	6 (13)	2 (17)	0.09
Lactate on ICU admission, mmol/l	1.6 (1.1, 2.3)	1.7 (1.2, 2.7)	1.2, (1.0, 2.0)	0.06
Hematocrit on ICU admission, %	27 (25, 31)	27 (25, 29)	32 (27, 35)	0.07
Alkalosis on ICU admission^a^, n (%)	7 (12)	7 (15)	0	
Catecholamine infusion on ICU admission, n (%)	23 (38)	21 (46)	2 (14)	0.06
Nutrition on ICU admission, n (%)	6 (10)	6 (13)	0	
Acute liver failure, n (%)	3 (5)	2 (4)	1 (7)	0.56
Chronic alcohol abuse, n (%)	4 (7)	3 (7)	1 (7)	1.00
Acute alcohol intoxication, n (%)	1 (2)	1 (2)	0	
ICU length of stay, days	3.7 (2.2, 6.2)	3.7 (2.3, 6.9)	3.4 (2.1, 3.8)	0.26
Hospital length of stay, days	11 (6.7, 24)	11 (7.2, 23)	13 (5.4, 24)	0.86
ICU mortality, n (%)	5 (8)	4 (9)	1 (7)	0.85
Hospital mortality, n (%)	6 (10)	5 (11)	1 (7)	0.68

Values are median (IQR) or n (%)

*BGL* blood glucose level, *BMI* body mass index, *APACHE* Acute Physiology and Chronic Health Evaluation, *ICU* intensive care unit

^a^pH >7.45

### Glycemic control, ketonemia and ketonuria

We analysed 280 blood glucose measurements, 277 blood ketone measurements, 261 urine ketone measurements and 276 hematocrit measurements obtained within 8.3 (1.6, 12.0) hours after ICU admission and daily until ICU discharge or day 10, whichever came first. A total of 274 (99 %) hematocrit levels were below normal (<40 %). Furthermore, hematocrit decreased significantly over a maximum observation period of 10 days in ICU (Additional file 1: Figure S1).

Overall, 27 of 46 (59 %) patients with a peak BGL ≥10 mmol/l required insulin to maintain their BGLs between 10 and 14 mmol/l. Of these 27 patients, 74 % had preexisting insulin-dependent diabetes. In addition, 27 of 46 (59 %) patients with a peak BGL ≥10 mmol/l received enteral and/or parenteral nutrition during ICU admission whereas only two (14 %) patients with a peak BGL <10 mmol/l received such nutrition. Mean total caloric intake progressively increased during ICU admission (Additional file 1: Figure S2). Only one patient received parenteral nutrition, which did not contain medium-chain triglycerides (OliClinomel, Baxter Healthcare, Deerfield, IL, USA). Insulin was not administered to patients with a peak BGL <10 mmol/l in ICU. Half of all patients received catecholamine infusion (mainly noradrenaline) while in ICU (Table 2).

**Table 2:** Glycemic control, ketonemia, ketonuria and acid–base status of diabetic patients with and without permissive hyperglycemia

Variable	All patients	Peak BGL	Peak BGL	*P* value
		≥10 mmol/l	<10 mmol/l	
Number of patients, n (%)	60 (100)	46 (77)	14 (23)	
No. of blood glucose measurements, n (%)	280	229	51	
No. of blood glucose measurements per patient	4 (3, 7)	4 (3, 7)	3 (2, 5)	0.19
Peak glucose level, mmol/l	12.0 (10.0, 15.0)	14 (12, 17)	8.2 (7.4, 9.5)	<0.001
Mean glucose level, mmol/l	10.0 (8.3, 13.0)	12 (9.5, 13)	7.1 (6.6, 8.3)	<0.001
Min glucose level, mmol/l	8.0 (6.2, 9.4)	8.8 (7, 9.9)	6.2 (5.7, 7.5)	0.007
No. of blood ketone measurements, n (%)	277	228	49	
No. of blood ketone measurements per patient	4 (3, 7)	4 (3, 7)	3 (2, 4)	0.11
Time between ICU admission until first measurement, hours	8.3 (1.6, 12)	8.9 (1.7, 13)	3.2 (1.5, 11)	0.02
Patients receiving insulin, n (%)	27 (45)	27 (59)	0	
Intravenous insulin rate, units/h	2 (1, 2)	2 (1, 2)	0	
Patients receiving nutrition, n (%)	29 (48)	27 (59)	2 (14)	
Enteral nutrition, n (%)	28 (47)	26 (57)	2 (14)	
Total parenteral nutrition, n (%)	2 (3.3)	2 (4.3)	0	
Patients receiving catecholamine infusion, n (%)	30 (50)	27 (46)	3 (21)	0.03
Noradrenaline, n (%)	29 (48)	26 (57)	3 (21)	0.03
Maximum noradrenaline rate, μg/min	5.0 (2.0, 16)	7.5 (2.0, 16)	2.0 (1.0, 4.0)	0.11
Adrenaline, n (%)	7 (12)	7 (15)	0	
Maximum adrenaline rate, μg/min	3.0 (2.0, 4.0)	3.0 (2.0, 4.0)	NA	
Ketosis episodes >0.6 mmol/l, n (%)	79 (28.6)	63 (28)	16 (33)	0.43
Peak blood ketones, mmol/l	0.8 (0.3, 1.9)	0.85 (0.2, 1.9)	0.65 (0.3, 1.0)	0.32
Worst ketosis, n (%)				0.52
No ketosis (<0.6 mmol/l)	22 (36.7)	16 (35)	6 (42)	
Mild (0.6–1.5 mmol/l)	20 (33.3)	15 (33)	5 (36)	
Moderate (1.6–3.0 mmol/l)	11 (18.3)	8 (17)	3 (21)	
Marked (>3.0 mmol/l)	7 (11.7)	7 (15)	0	
Number of urine ketone measurements	261	215	46	
Worst ketonuria, n (%)				0.51
No ketonuria	40 (66.7)	31 (67)	9 (64)	
Mild (1+)	13 (21.7)	10 (22)	3 (21)	
Moderate (2+)	6 (10)	5 (11)	1 (7)	
Marked (3+)	1 (1.7)	0	1 (7)	
Patients with alkalosis^a^, n (%)	25 (42)	20 (43)	5 (36)	0.76
Blood gas analysis, *worst value during study period*				
pH	7.4 (7.3, 7.4)	7.3 (7.3, 7.4)	7.4 (7.3, 7.4)	0.32
Lactate, mmol/l	2.2 (1.6, 3.1)	2.3 (1.7, 3.4)	2 (1.4, 2.3)	0.15
Bicarbonate, mmol/l	22 (20, 24)	22 (19, 24)	25 (23, 26)	0.002
Chloride, mmol/l	108 (104, 111)	108 (104, 111)	105 (102, 108)	0.10
Sodium, mmol/l	136 (132, 138)	136 (132, 138)	136 (132, 136)	0.73
Albumin, g/l	26 (22, 30)	26 (21, 29)	29 (23, 30)	0.75
Base excess (BE), mmol/l	−2.8 (−5, 0)	−3 (−5, −1)	0.35 (−4, 1)	0.006
ADA ketoacidosis, n (%)	2/60 (3.3)	1/46 (2.2)	1/14 (7.1)	1.00
JBDS ketoacidosis, n (%)	0/60 (0)			

Values are median (IQR) or n (%)

*BGL* blood glucose level, *ICU* intensive care unit, *ADA* American Diabetes Association, *JBDS* Joint British Diabetes Society

^a^pH >7.45

Overall, 38 (63 %) patients showed some degree of ketosis (β-OHB ≥0.6 mmol/l) during ICU admission (20 [33 %] mild, 11 [18 %] moderate and seven [12 %] severe ketosis). A total of 20 (33 %) patients had some degree of ketonuria (13 [22 %] mild, six [10 %] moderate and one [2 %] severe ketonuria). Additionally, 25 (42 %) patients had at least one episode of alkalosis (pH >7.45). On crude comparison, we observed a similar prevalence and severity of ketosis and ketonuria in patients with peak BGL above and below 10 mmol/l. In contrast, patients with a peak BGL ≥10 mmol/l had lower bicarbonate levels (*P* = 0.001) and base excess (*P* = 0.006).

However, only two out of 60 (3.3 %) patients (one patient in each group) fulfilled the criteria for ketoacidosis according to the JBDS consensus criteria (no patients developed ADA-defined ketoacidosis, Table 2). A description of these two individuals is provided in the Additional file 1.

Patients with (n = 38) and without (n = 22) ketosis had similar HbA1c levels (median HbA1c 6.7 % in both groups, *P* = 0.72) whereas admission hematocrit levels were higher in patients with ketosis (*P* = 0.046). A similar proportion of patients had insulin-dependent diabetes in the two groups (42 vs. 50 %). Moreover, admission and peak BGLs did not differ significantly (Additional file 1: Table S1 and S2).

In contrast, compared to patients with mild to moderate ketosis (n = 31), insulin-dependent diabetes tended to be more common (71 % vs. 35 %, *P* = 0.12), HbA1c was significantly greater (8.2 % vs. 6.5 %, *P* <0.001) and admission hematocrit was lower (25 % vs. 30 %, *P* = 0.03) among patients with severe ketosis (n = 7) (Additional file 1: Table S3).

**Fig. 1:**
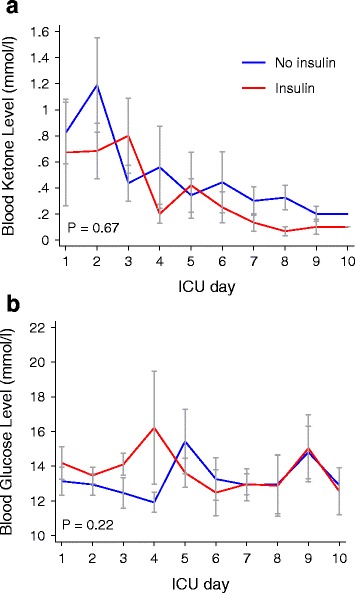
Mean (SEM) blood ketone levels (**a**) and blood glucose levels (**b**) in patients with (*red line*) and without (*blue line*) insulin therapy. Only patients with a blood glucose level above 10 mmol/l at each time point included. *P* values represent the between-group comparison on repeated-measures analysis of variance

In patients with BGL ≥10 mmol/l, ketosis resolved within 3–4 days irrespective of whether insulin was administered or not (Fig. 1a) and despite persistent hyperglycemia according to protocol (Fig. 1b).

**Fig. 2:**
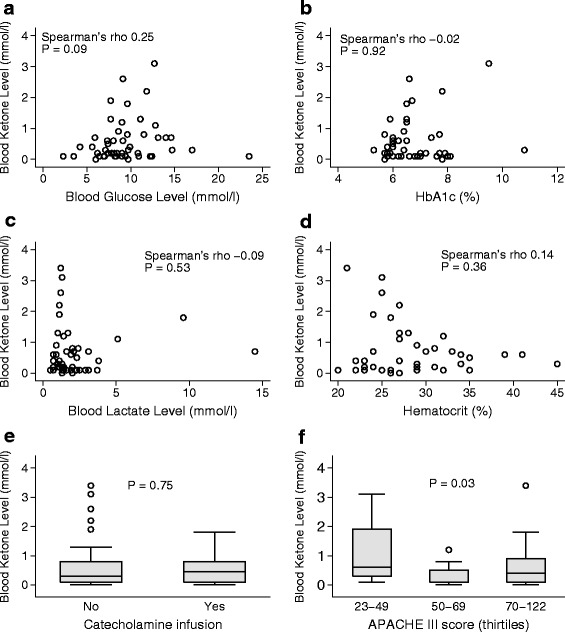
Correlation between blood ketone levels and blood glucose levels (**a**), HbA1c (**b**), lactate (**c**), hematocrit (**d**), catecholamine infusion (**e**) and APACHE III quartiles (**f**) on ICU admission in patients not receiving insulin

We found no significant correlation between admission blood ketone levels and admission BGLs (Fig. 2a), HbA1c levels (Fig. 2b), blood lactate levels (Fig. 2c), hematocrit levels (Fig. 2d), or catecholamine infusion (Fig. 2e) in patients not receiving insulin (n = 43). In contrast, patients in the mid-APACHE III tertile had lower admission blood ketone levels than patients in the highest and lowest tertile (Fig. 2f). Patients with alkalosis on admission had significantly lower blood ketone levels than patients without alkalosis (*P* = 0.04) whereas patients admitted following cardiopulmonary bypass surgery had significantly greater blood ketone levels (median [IQR] 0.6 [0.2, 1.3] mmol/l vs. 0.2 [0.1, 0.7] mmol/l than patients without cardiopulmonary bypass surgery, *P* = 0.049). On multivariable linear regression analysis, restricted to patients not receiving insulin (n = 43), we observed no independent association between blood ketone levels and blood glucose levels, HbA1c levels, blood lactate levels, hematocrit levels, catecholamine infusion or APACHE III score. However, a trend towards greater blood ketone levels in patients admitted following cardiopulmonary bypass was observed in the adjusted analysis (*P* = 0.06) (Table 3).

**Table 3:** Multivariable linear regression analysis showing the association with admission blood ketone level (mmol/l) in patients not receiving insulin

	Univariable analysis		Multivariable analysis	
Variable	estimate (95 % CI)	*P* value	estimate (95 % CI)	*P* value
Blood glucose level, per mmol/l	0.03 (−0.04 to 0.10)	0.35	0.03 (−0.05 to 0.12)	0.44
HbA1c, per %	0.16 (−0.08 to 0.40)	0.18	0.16 (−0.10 to 0.42)	0.21
Blood lactate, per mmol/l	0.02 (−0.08 to 0.12)	0.72	0.05 (−0.08 to 0.19)	0.41
Hematocrit, per %	−0.02 (−0.06 to 0.03)	0.38	−0.03 (−0.08 to 0.01)	0.15
Catecholamine infusion				
No	Referent		Referent	
Yes	−0.19 (−0.67 to 0.29)	0.43	−0.57 (−1.30 to 0.16)	0.12
Cardiopulmonary bypass surgery				
No	Referent		Referent	
Yes	0.44 (−0.02 to 0.91)	0.06	0.67 (−0.03 to 1.37)	0.06
APACHE III score (tertile)				
23–49	Referent		Referent	
50–69	−0.68 (−1.23 to −0.13)	0.02	−0.41 (−1.14 to 0.33)	0.27
70–122	−0.30 (−0.85 to 0.25)	0.28	−0.29 (−0.54 to 1.12)	0.49

*HbA1c* glycated hemoglobin A1c, *APACHE* Acute Physiology and Chronic Health Evaluation

## Discussion

### Key findings

We performed a prospective observational study to explore the prevalence of ketonemia and ketonuria in critically ill diabetic patients treated within a liberal glycemic control protocol. Overall, about two thirds of our patients had some degree of ketosis (β-OHB ≥0.6 mmol/l) during their ICU stay and 33 % had some degree of ketonuria. However, ketosis in these critically ill diabetic patients managed with liberal glycemic control was typically seen in the first 24–48 hours of admission and appeared independent of glucose control, HbA1c level and severity of illness. Moreover, ketosis resolved in all patients irrespective of whether insulin was given or not. Finally, ketosis appeared more pronounced following cardiopulmonary bypass surgery.

### Relationship to previous studies

To our knowledge, this is the first study investigating the prevalence of ketosis in critically ill diabetic patients without acute diabetic ketoacidosis or hyperosmolar hyperglycemic state. We are also the first to describe the prevalence of ketosis in patients treated with a liberal glucose protocol (BGL target 10–14 mmol/l).

In accordance with our results, a study of 32 ventilated non-diabetic ICU patients with (n = 19) and without (n = 13) stress hyperglycemia (BGL up to 16 mmol/l) found no relationship between breath acetone concentration and BGL [17]. While in most patients (n = 11) breath acetone concentration decreased after insulin administration, some had persistent ketosis despite normal BGL.

The two main ketone bodies, acetoacetate (AcAc) and 3-beta-hydroxybutyrate (β-OHB), usually circulate in low concentrations (<0.5 mmol/l) in healthy adults [18]. Serum levels of β-OHB start to rise after 12–16 hours of fasting (an essentially ubiquitous state in critically ill patients in the period surrounding ICU admission), reaching 1–2 mmol/l after 2 days [19–21], and 6–8 mmol/l after 2–3 weeks of starvation [22, 23].

Harano et al. [13] compared serum ketone levels between non-acutely ill type 1 (n = 117) and type 2 (n = 260) diabetic patients and 91 healthy subjects after an overnight fast. In patients with type 1 diabetes, ketone bodies were higher despite normal fasting BGL and increased proportionally to the BGL in hyperglycemic individuals. In type 2 diabetics, however, the authors found only a weak correlation between fasting plasma glucose and ketonemia with serum ketone levels rarely exceeding 2 mmol/l. Interestingly, a post-prandial reduction in 3-hydroxybutyrate was noted in subjects with a sufficient endogenous insulin response (on dietary control or sulphonylureas), but not in subjects with insulin-dependent type 2 diabetes, who are also known to have greater ketogenesis after nighttime fasting (12). Duska et al. [24] explored the relative impact of exogenous insulin administration and endogenous insulin secretion on plasma insulin levels in critically ill trauma patients without diabetes. They found that, in such patients without beta-cell dysfunction, the plasma insulin level is mainly determined by the endogenous secretion rather than by the insulin infusion, even during the acute phase when exogenous insulin requirements were high.

Preserved beta-cell function with sufficient endogenous insulin secretion could also explain the relatively low blood ketone levels in our cohort and the similar ketone kinetics in hyperglycemic patients with and without exogenous insulin administration. Moreover, among the few patients with marked ketonemia (n = 7) the majority was insulin-dependent suggesting significant beta-cell failure. However, even in this subgroup ketonemia resolved despite permissive hyperglycemia.

The effect of fasting on urine ketone concentration has been studied in a large Korean trial of 16,523 volunteers [25]. Only 8.8 % of all subjects developed ketonuria after fasting overnight. Among those *without* ketonuria, the prevalence of obesity and metabolic syndrome was significantly higher and cholesterol levels, insulin levels and body mass index (BMI) were more likely to be abnormal. A ketotic response to fasting could, therefore, indicate a favorable metabolic state.

In addition to fasting, ketosis (and ketoacidosis) may be caused by excessive alcohol consumption, severe hypoxia, counter-regulatory hormone excess, catecholamine infusion, parenteral nutrition, alkalosis, severe liver disease, and various inborn errors of metabolism [8, 9, 11, 18, 26, 27]. However, among our patients with acute liver failure (n = 3), chronic alcohol intake (n = 4) and acute alcohol intoxication (n = 1) only one patient (acute-on-chronic liver failure) developed ketosis (peak blood β-OHB level 1.4 mmol/l). Furthermore, catecholamine infusion or alkalosis was not associated with elevated β-OHB levels in our study.

In contrast, in our patients, cardiopulmonary bypass surgery (performed during normothermia) appeared to be an important trigger of ketogenesis. This finding is supported by Hashimoto et al. [12]. In 24 patients, they compared the arterial acetoacetate to β-OHB ratio (the so-called ketone body ratio) between patients undergoing normothermic vs. hypothermic cardiopulmonary bypass surgery. The ratio decreased significantly in both groups suggesting reduced mitochondrial redox potential favoring conversion to β-OHB in such patients. Importantly, the decrease in ketone body ratio was more pronounced after hypothermic bypass indicating even greater β-OHB generation in that group.

### Implications of study findings

Our finding that a BGL ≥10 mmol/l is not associated with increased ketogenesis and that critical illness is associated with some degree of peri-admission ketosis irrespective of blood glucose level and insulin therapy implies that a BGL range of 10–14 mmol/l in critically ill diabetic patients may be associated with minimal additional ketogenic metabolic stress.

Low nutritional intake in the hours and days before ICU admission is a plausible explanation for the observed early ketosis of critical illness in our population. Indeed, although only about half of our patients received nutrition, their caloric intake progressively increased, which may have contributed to the observed decline in ketone levels over time. In addition, it seems that endogenous insulin secretion due to residual beta-cell function may be sufficient to suppress ketogenesis in patients not requiring insulin. This hypothesis is also supported by a lack of correlation between the level of ketonemia and blood glucose in our patients. In addition, even in our patients with premorbid insulin-dependent diabetes, the majority of which also required insulin in ICU, early ketosis resolved despite sustained hyperglycemia.

The clinical relevance of ketosis in ICU patients remains unclear as recent studies suggest that β-OHB could have anti-inflammatory effects by inhibiting interleukin (IL)-1β and IL-18 production [28]. In addition, ketone bodies provide important energy source for vital organs not only during starvation but also during stress-induced insulin resistance. Ketogenesis could, therefore, be an adaptive and protective response to critical illness.

### Strengths and limitations

Our study has several strengths. We prospectively analyzed a representative sample of patients with diabetes using the same point-of-care analysis platform in every patient. The setting of a tertiary hospital with a heterogeneous case mix is likely to reflect the average ICU population. We performed daily ketone measurements and simultaneously tested urine samples for ketonuria during the entire ICU stay or up to 10 consecutive days. We used a multivariable model to assess the relationship between blood ketone levels and glycemic control, markers of disease severity and insulin administration. Our findings are robust and demonstrate that blood ketone levels are unrelated to blood glucose levels or insulin treatment in the critically ill diabetic patient without diabetic ketoacidosis within the boundaries of a liberal glucose protocol.

Our study has some limitations. First, it is observational in nature and not a randomized controlled study. However, our aim was to explore the prevalence of ketosis during liberal glucose management of critically ill diabetic patients. Second, this is a single-center study with only a limited number of patients. However, we used solid methodology and obtained more than 270 blood ketone measurements and analyzed more than 100 hyperglycemic episodes. Third, we used a semi-quantitative method to measure ketonuria. However, given the inherent limitations [18, 27] of point-of-care urine ketone tests, and in view of our results, it is unlikely that quantitative methods would have influenced our results. Yet, previous validation studies suggest that the use of point-of-care instruments may overestimate true blood β-OHB levels when hematocrit is below normal [14], which was the case in most of our patients. Hence, we may have overestimated the true ketosis prevalence. Fourth, we did not measure plasma insulin concentrations or C-peptide levels and were therefore not able to quantify beta-cell function. Furthermore, although we lack data on counter-regulatory hormone, FFA, amino acid and oxaloacetate levels, we assessed clinical variables relevant to ketogenesis. Fifth, we did not measure glycosuria, which may be particularly pronounced in critically ill patients during permissive hyperglycemia. The degree of glycosuria in such patients and its impact on patient-centered outcomes should therefore be explored in future studies. Sixth, we did not measure acetoacetate and acetone and could therefore not quantify the total ketotic activity. Perturbations in the mitochondrial redox state during critical illness may alter the ratio between acetoacetate and β-OHB [29]. However, we performed repeated β-OHB measurements over several days and observed a consistent and marked decline over time. It is unlikely that unmeasured ketones would have a different directional change over the corresponding time frame. Finally, because the results of the daily ketone measurements were documented on the observational chart, we cannot exclude that these data influenced glucose management by clinicians. However, nursing and research staff performed ketone measurements and documentation without direct involvement of medical staff.

## Conclusions

Ketosis in critically ill diabetic patients is common and typically occurs in the period early after admission. Ketosis may be particularly pronounced following cardiopulmonary bypass surgery. However, in critically ill diabetic patients, liberal glycemic control or insulin therapy does not appear to be associated with ketosis. These findings suggest that liberal glycemic control in these patients does not cause additional ketotic stress and imply that factors beyond glycemic control and insulin therapy may be more important drivers of ketosis in such patients.

## Additional file

Additional file 1: Table S1. Baseline characteristics of diabetic patients with and without ketosis. **Table S2.** Glycemic control in diabetic patients with and without ketosis. **Table S3.** Baseline characteristics of patients with mild or moderate and marked ketosis. Description of the two patients with American Diabetes Association (ADA) ketoacidosis. **Figure S1.** Change in hematocrit over time for all study patients. **Figure S2.** Daily mean caloric intake in patients receiving nutrition. (DOCX 90 kb)

## Abbreviations

ABG: arterial blood gas
AcAc: acetoacetate
ADA: American Diabetes Association
APACHE: Acute Physiology and Chronic Health Evaluation
BGL: blood glucose level
β-OHB: 3-beta-hydroxybutyrate
DKA: diabetic ketoacidosis
FFA: free fatty acids
HbA1c: glycated hemoglobin A1c
HHS: hyperglycemic hyperosmolar state
ICU: intensive care unit
IL: interleukin
JBDS: Joint British Diabetes Society
RM-ANOVA: repeated-measures analysis of variance
RRT: renal replacement therapy
