# Supplementary material for: Prevalence of ketosis, ketonuria, and ketoacidosis during liberal glycemic control in critically ill patients with diabetes: an observational study
Source: Crit Care. 2016 Sep 15;20:297. doi: 10.1186/s13054-016-1462-7 (PMC5025567; doi:10.1186/s13054-016-1462-7)
Supplement: Additional file 1: Table S1. — Baseline characteristics of diabetic patients with and without ketosis. Table S2. Glycemic control in diabetic patients with and without ketosis. Table S3. Baseline characteristics of patients with mild or moderate and marked ketosis. Description of the two patients with American Diabetes Association (ADA) ketoacidosis. Figure S1. Change in hematocrit over time for all study patients. Figure S2. Daily mean caloric intake in patients receiving nutrition. (DOCX 90 kb) [file 13054_2016_1462_MOESM1_ESM.docx]

Additional file 1

Table S1. Baseline characteristics of diabetic patients with and without ketosis

|  | All patients | Ketosis | No Ketosis | P value |
| --- | --- | --- | --- | --- |
| Number of patients | 60 | 38 | 22 |  |
| Age, years | 66 (60, 74) | 67 (59, 73) | 65 (60, 77) | 0.95 |
| Female sex, n (%) | 17 (28) | 12 (32) | 5 (23) | 0.34 |
| Weight, kg (n=58) | 84 (74, 100) | 82 (71, 104) | 86 (79, 96) | 0.89 |
| APACHE III score | 60 (46, 76) | 56 (44, 77) | 61 (48, 69) | 0.98 |
| HbA1c on ICU admission, % | 6.7 (6.0, 7.7) | 6.7 (6.0, 7.6) | 6.7 (6.0, 7.9) | 0.72 |
| Blood ketones on ICU admission, mmol/l | 0.3 (0.1, 0.8) | 0.7 (0.2. 1.3) | 0.1 (0.1, 0.2) | <0.001 |
| Lactate on ICU admission, mmol/l | 1.6 (1.1, 2.3) | 1.6 (1.1, 2.2) | 1.6 (1.3, 2.5) | 0.63 |
| Blood glucose on ICU admission, mmol/l | 9.3 (7.6, 12) | 9.1 (7.7, 13.0) | 9.6 (7.1, 12.0) | 0.71 |
| Hematocrit on ICU admission, % | 27 (25, 31) | 29 (25, 33) | 27 (23, 29) | 0.046 |
| Alkalosis on ICU admission, n (%) | 7 (12) | 4 (11) | 3 (14) | 0.70 |
| Catecholamine infusion on ICU admission, n (%) | 23 (38) | 16 (42) | 7 (32) | 0.58 |
| Nutrition on ICU admission, n (%) | 6 (10) | 4 (11) | 2 (9) | 1.0 |
| Acute liver failure, n (%) | 3 (5) | 1 (3) | 2 (9) | 0.55 |
| Chronic alcohol abuse, n (%) | 4 (7) | 0 | 4 (18) | 0.02 |
| Acute alcohol intoxication, n (%) | 1 (2) | 0 | 1 (5) | 0.37 |
| Type I diabetes, n (%) | 3 (5) | 2 (5) | 1 (5) | 1.00 |
| Type II diabetes, n (%) | 57 (95) | 35 (92) | 21 (95) | 1.00 |
| Type II diabetes treatment, n (%) |  |  |  | 0.30 |
| Diet only | 8 (13) | 5 (13) | 6 (27) |  |
| Oral agent(s) only | 25 (42) | 19 (50) | 6 (27) |  |
| Insulin only | 11 (18) | 6 (16) | 5 (23) |  |
| Insulin + oral agent(s) | 13 (22) | 8 (21) | 5 (23) |  |
| Non-operative admission diagnosis, n (%) |  |  |  | 0.55 |
| Cardiovascular | 6 (10) | 4 (29) | 2 (16) |  |
| Sepsis | 6 (10) | 3 (21) | 3 (25) |  |
| Respiratory | 6 (10) | 4 (29) | 2 (17) |  |
| Renal/Metabolic | 6 (10) | 2 (14) | 4 (33) |  |
| Other | 2 (3) | 1 (7) | 1 (8) |  |
| Operative admission diagnosis, n (%) |  |  |  | 0.71 |
| Cardiovascular | 25 (42) | 18 (75) | 7 (70) |  |
| Gastrointestinal | 6 (10) | 4 (17) | 2 (20) |  |
| Other | 3 (5) | 2 (8) | 1 (10) |  |

Table S2. Glycemic control in diabetic patients with and without ketosis

|  | All patients | Ketosis | No Ketosis | P value |
| --- | --- | --- | --- | --- |
| Number of patients, n (%) | 60 (100) | 38 (63) | 22 (37) |  |
| Number of glucose measurements | 280 | 197 | 83 |  |
| Peak glucose level, mmol/l | 12.0 (10.0, 15.0) | 13 (11, 17) | 11 (9.7, 14) | 0.07 |
| Mean glucose level, mmol/l | 10.0 (8.3, 13.0) | 11 (8.4, 13) | 9.8 (8.1, 11) | 0.25 |
| Min glucose level, mmol/l | 8.0 (6.2, 9.4) | 7.8 (5.9, 9.7) | 8.4 (6.2, 9.4) | 0.99 |
| Time in target range, % | 33 (0, 54) | 33 (0, 60) | 27 (0, 50) | 0.50 |
| Time above target range, % | 0 (0, 27) | 0 (0, 30) | 0 (0, 14) | 0.21 |
| Time below target range, % | 50 (15, 88) | 40 (10, 86) | 54 (25, 100) | 0.33 |
| Episodes with BG > 14mmol/l, n (%) | 48 (17.1) | 40 (20.3) | 8 (9.6) | 0.04 |
| Episodes with BG < 10mmol/l, n (%) | 133 (47.5) | 85 (43.2) | 48 (57.8) | 0.03 |
| Absolute hypoglycemia episodes, n (%) | 3 (1.1) | 0 | 3 (3.6) | 0.03 |
| Patients receiving nutrition, n (%) | 29 (48) | 20 (53) | 9 (41) | 0.38 |
| Enteral nutrition, n (%) | 28 (47) | 19 (50) | 9 (41) | 0.50 |
| Total parenteral nutrition, n (%) | 2 (3.3) | 1 (2.6) | 1 (4.5) | 1.0 |
| Patients receiving catecholamine infusion, n (%) | 30 (50) | 21 (55) | 9 (41) | 0.28 |
| Noradrenaline, n (%) | 29 (48) | 21 (55) | 8 (36) | 0.16 |
| Maximum noradrenaline rate, µg/min | 5.0 (2.0, 16) | 8.0 (2.0, 18) | 3.0 (1.5, 8.5) | 0.11 |
| Adrenaline, n (%) | 7 (12) | 5 (13) | 2 (9) | 1.0 |
| Maximum adrenaline rate, µg/min |  |  |  |  |
| Acute CRRT, n (%) | 12 (20) | 8 (21) | 4 (18) | 1 |
| Peak Blood Ketones, mmol/l | 0.8 (0.3, 1.9) | 1.4 (0.8, 2.6) | 0.2 (0.2, 0.3) | <0.001 |
| Mean Blood ketones, mmol/l | 0.3 (0.2, 0.8) | 0.6 (0.4, 1.1) | 0.2 (0.1, 0.2) | <0.001 |
| Worst ketosis, n (%) |  |  |  | <0.001 |
| No ketosis (<0.6) | 22 (36.7) | 0 | 22 (100) |  |
| Mild (0.6-1.5) | 20 (33.3) | 20 (52.6) | 0 |  |
| Moderate (1.6-3.0) | 11 (18.3) | 11 (28.9) | 0 |  |
| Marked (>3.0) | 7 (11.7) | 7 (18.4) | 0 |  |
| Number of urine ketone measurements | 261 | 180 | 81 |  |
| Worst ketonuria, n (%) |  |  |  | 0.13 |
| No ketonuria | 40 (66.7) | 22 (57.9) | 18 (81.8) |  |
| Mild (1+) | 13 (21.7) | 9 (23.6) | 4 (18.2) |  |
| Moderate (2+) | 6 (10) | 6 (15.8) | 0 |  |
| Marked (3+) | 1 (1.7) | 1 (2.6) | 0 |  |
| ADA ketoacidosis, n (%) | 2/60 (3.3) | 1 | 0 | 1 |
| ADA episodes, n (%) | 2/272 (0.7) |  |  |  |
| JBDS ketoacidosis, n (%) | 0/60 (0) | 0 | 0 | 1 |
| Insulin episodes, n (%) | 69 (24.7) | 54 (27.3) | 15 (18) | 0.13 |
| Insulin episodes per patient, n | 0 (0, 2) | 0 (0, 2) | 0 (0, 1) | 0.26 |
| Patients with alkalosis, n (%) | 25 (42) | 17 (45) | 8 (36) | 0.53 |
| Blood gas analysis |  |  |  |  |
| pH | 7.4 (7.4, 7.4) | 7.4 (7.4, 7.4) | 7.4 (7.4, 7.4) | 0.67 |
| Lactate, mmol/l | 1.6 (1.2, 2.1) | 1.7 (1.4, 2.2) | 1.4 (1.2, 1.7) | 0.18 |
| Bicarbonate, mmol/l | 26 (23, 27) | 26 (23, 28) | 25 (22, 27) | 0.22 |
| Chloride, mmol/l | 104 (101, 107) | 103 (101, 107) | 104 (101, 108) | 0.29 |
| Sodium, mmol/l | 137 (135, 140) | 137 (135, 141) | 138 (136, 140) | 0.90 |
| Albumin, g/l | 28 (23, 32) | 29 (26, 32) | 27 (22, 32) | 0.40 |
| Base excess (BE), mmol/l | 1.5 (0, 3.6) | 0.9 (-1.5, 3) | 1 (-1.9, 3) | 0.68 |

Values are median (IQR) or number (%)

Table S3. Baseline characteristics of patients with mild or moderate and marked ketosis

|  | All patients | Marked Ketosis | Mild/moderate Ketosis | P value |
| --- | --- | --- | --- | --- |
| Number of patients | 60 | 7 | 31 |  |
| Age, years | 66 (60, 74) | 68 (54, 76) | 66 (59, 73) | 0.87 |
| Female sex, n (%) | 17 (28) | 2 (29) | 10 (32) | 0.62 |
| Weight, kg (n=58) | 84 (74, 100) | 79 (63, 98) | 88 (73, 112) | 0.17 |
| APACHE III score | 60 (46, 76) | 48 (40, 80) | 64 (44, 77) | 0.68 |
| HbA1c on ICU admission, % | 6.7 (6.0, 7.7) | 8.2 (7.7, 9.5) | 6.5 (6.0, 7.2) | 0.001 |
| Blood ketones on ICU admission, mmol/l | 0.3 (0.1, 0.8) | 2.2 (0.2, 3.1) | 0.6 (0.2, 1.1) | 0.17 |
| Lactate on ICU admission, mmol/l | 1.6 (1.1, 2.3) | 1.2 (0.7, 2.0) | 1.7 (1.1, 2.4) | 0.13 |
| Blood glucose on ICU admission, mmol/l | 9.3 (7.6, 12) | 9.5 (7.8, 13.0) | 9.1 (7.7, 13.0) | 0.75 |
| Hematocrit on ICU admission, % | 27 (25, 31) | 25 (23, 28) | 30 (27, 34) | 0.03 |
| Alkalosis on ICU admission, n (%) | 7 (12) | 0 | 4 (13) |  |
| Catecholamine infusion on ICU admission, n (%) | 23 (38) | 2 (29) | 14 (45) | 0.68 |
| Nutrition on ICU admission, n (%) | 6 (10) | 0 | 4 (14) |  |
| Acute liver failure, n (%) | 3 (5) | 0 | 1 (3) | 1.00 |
| Chronic alcohol abuse, n (%) | 4 (7) | 0 | 0 |  |
| Acute alcohol intoxication, n (%) | 1 (2) | 0 | 0 |  |
| Type-1 diabetes, n (%) | 3 (5) | 0 | 2 (6) | 1.00 |
| Type-2 diabetes, n (%) | 57 (95) | 7 (100) | 28 (90) | 1.00 |
| Type-2 diabetes treatment, n (%) |  |  |  | 0.12 |
| Diet only | 8 (13) | 0 | 5 (16) |  |
| Oral agent(s) only | 25 (42) | 2 (29) | 17 (55) |  |
| Insulin only | 11 (18) | 3 (43) | 3 (10) |  |
| Insulin + oral agent(s) | 13 (22) | 2 (29) | 6 (19) |  |
| Non-operative admission diagnosis, n (%) |  |  |  | 1.00 |
| Cardiovascular | 6 (10) | 1 (100) | 3 (23) |  |
| Sepsis | 6 (10) | 0 | 3 (23) |  |
| Respiratory | 6 (10) | 0 | 4 (31) |  |
| Renal/Metabolic | 6 (10) | 0 | 2 (15) |  |
| Other | 2 (3) | 0 | 1 (8) |  |
| Operative admission diagnosis, n (%) |  |  |  | 0.75 |
| Cardiovascular | 25 (42) | 6 (100) | 12 (67) |  |
| Gastrointestinal | 6 (10) | 0 | 4 (22) |  |
| Other | 3 (5) | 0 | 2 (11) |  |

Values are medians (IQR) or number (%)

Description of the two patients with American Diabetes Association (ADA) ketoacidosis:

Patient 1: 57 years old male, admitted to ICU after elective liver transplantation for NASH, T2DM on OHG. Prolonged and complicated operation (OT > 12hours), fulfilled ADA ketoacidosis criteria upon ICU admission. ABG on ICU admission: pH 7.20, bicarbonate 12mmol/l, lactate 13 mmol/l.

Patient 2: 69 years old female, septic shock with development of rapid multi-organ failure, severe hyperglycemia and metabolic acidosis on day 4.

Figure S1. Mean (SEM) hematocrit levels in all study patients (n=60). P-value represent the assessment of change over time on repeated measures analysis of variance.

Figure S2. Daily mean caloric intake (enteral + parenteral) in patients receiving nutrition (n=29).
